# Family impact of Rotavirus Gastroenteritis in Taiwan and Vietnam: an Ethnographic Study

**DOI:** 10.1186/s12879-015-0968-y

**Published:** 2015-06-23

**Authors:** Megan A. O’Brien, Sònia Rojas-Farreras, Hung-Chang Lee, Lung-Huang Lin, Chieh-Chung Lin, Phuc Le Hoang, Montse Pedros, Núria Lara

**Affiliations:** Merck & Co., Inc., Whitehouse Station, NJ USA; IMS Health, Barcelona, Spain; Department of Pediatrics, MacKay Memorial Hospital, Hsinchu, Taiwan; Department of Pediatrics, Cathay General Hospital, Taipei, Taiwan & School of Medicine, Fu-Jen Catholic University, New Taipei City, Taiwan; Department of Pediatrics, Taichung Veterans General Hospital, Taichung, Taiwan; Children’s Hospital # 1, Ho Chi Minh City, Vietnam; IMS Health, Doctor Ferran 25-27 2°, 08034 Barcelona, Spain

**Keywords:** Rotavirus infection, Asia, Taiwan, Vietnam, Family impact, Ethnographic study, Focus groups, Economic impact, Emotional impact

## Abstract

**Background:**

Prior to the introduction of rotavirus vaccines, rotavirus was the leading cause of severe gastroenteritis in infants and young children, and it continues to be the leading cause in countries without vaccination programs. Rotavirus gastroenteritis results in substantial economic burden and has a pronounced effect on the family of those who are ill. Both in Taiwan and in Vietnam, rotavirus illness is viewed as a priority disease. This study assessed, in Taiwan and Vietnam, the impact of rotavirus gastroenteritis on the family among a group of parents whose children had recently been hospitalized for this illness.

**Methods:**

In the first half of 2013, parents of children who had been hospitalized due to rotavirus infection were recruited from hospitals in Taiwan (*n* = 12) and Vietnam (*n* = 22), and participated in focus group sessions or in-depth ethnographic interviews.

**Results:**

In both countries, the results point to a substantial burden on the parents concerning emotions and logistics of daily tasks, and to considerable disruptions of the family routine. Taiwanese parents reported satisfaction with the health care system, a great deal of effort to suppress emotions, a fair amount of knowledge about rotavirus, and little extra costs related to the illness. On the other hand, parents in Vietnam expressed concern about the emotional well-being of and the health care treatments for their children, were less knowledgeable regarding rotavirus infection, and experienced a substantial financial burden due to indirect costs that were related to accessing treatment.

**Conclusions:**

Families in Taiwan and Vietnam suffer from a considerable economic and emotional burden related to rotavirus gastroenteritis. One way to substantially reduce this burden is to provide universal and affordable rotavirus vaccination to susceptible children, especially since cost-effectiveness studies have demonstrated that universal vaccination would be safe and efficacious against severe rotavirus gastroenteritis in these countries.

## Background

Rotavirus is a leading cause of severe gastroenteritis in infants and young children, especially in developing countries: among children under five years of age, rotavirus accounts for about half a million deaths annually (29 % of all deaths due to diarrhea and 5 % of all deaths) [1–4]. Symptoms of rotavirus gastroenteritis (RGE) develop after an incubation period of two to 3 days and persist for several days. The disease is characterized by the sudden onset of watery diarrhea, fever, and vomiting, and is many times indistinguishable from other acute diarrheal illnesses. While most rotavirus infected children have only mild symptoms, 1.5–3 % of them develop severe RGE and dehydration that requires hospitalization and aggressive rehydration [2]. Severe dehydration following rotavirus diarrhea can lead to death.

A systematic review by Kawai et al. [5] on the burden of rotavirus gastroenteritis in Asia found on the basis of 113 eligible articles that the incidence rate of rotavirus-related hospitalizations among children under the age of five ranged from 2.1 to 20.0 cases per 1000 children per year. The study showed that the highest proportions of hospitalizations due to rotavirus were reported in Eastern Asia and South East Asia—Taiwan and Vietnam were identified among the five countries with the highest incidence rates. In these countries, rotavirus illness is detected year round [5–7]. In Taiwan, most of the RGE cases occur during the winter and the spring seasons [8, 9]: one study found an increase during the winter season from an overall 45 % rotavirus prevalence of fecal samples from children under age five in hospitals and 14 % in clinics to 60 and 21 %, respectively [10]. In Vietnam, most cases are detected during the months of September through December [11]. It has been estimated that as much as over 600,000 individuals with rotavirus illness seek medical care in Taiwan in a given year, which equals to as much as up to half of all Taiwanese children under the age of five years [12]. Similarly, 55 % of stool samples were found to be rotavirus-positive in a research study over a 5-year period at six hospitals in Vietnam of children under five years old (*n* > 10,000) who were hospitalized with diarrhea [13]. This study estimated that 5300–6800 children under age five die of rotavirus infection each year in Vietnam, representing 8–11 % of all deaths in this age group. The Vietnamese National Institute for Hygiene and Epidemiology attributed approximately 820,000 clinic visits and 122,000–140,000 hospitalizations to rotavirus-related diarrhea in a birth cohort of 1,639,000 Vietnamese children based on surveillance data between 1998 and 2003 [14].

Rotavirus illness has a pronounced effect on the family of those who are ill. A study in the US among parents of rotavirus-positive children revealed the negative impact of rotavirus illness on the family in form of disruptions of daily tasks and psychological stress [15]. Given the international burden of RGE, further studies are needed in various geographical and cultural settings to understand the effect that rotavirus illness has on the family of those who fall ill. The aim of this study was to use qualitative research methods to assess how families were impacted by rotavirus gastroenteritis in Taiwan and Vietnam in terms of their experiences related to the onset of the illness and the medical care their children received, changes of family routines, the economic impact of the illness, their emotions and feelings during the illness, and their knowledge about rotavirus. In addition, the study also assessed the importance ranking of certain aspects of parents’ experience with the illness.

## Methods

### Study design and setting

The Family Impact of Rotavirus Gastroenteritis study was a qualitative study using focus groups and ethnographic in-depth interviews (based on a focus group/interview guide) that assessed the impact of rotavirus gastroenteritis of affected families in Taiwan and Vietnam. Recruitment for the study took place between February 2013 and June 2013 in Taipei, Taiwan and January 2013 and April 2013 in Ho Chi Minh City (HCMC) in Vietnam. Study investigators were pediatricians based at Mackay Memorial Hospital, Cathay General Hospital and Taichung Veterans General Hospital in Taiwan, and HCMC Central Pediatric Hospital in Vietnam, treating at least five children per month with suspected RGE. After investigators were trained on the study protocol and procedures, they identified children who might be eligible, and invited their parents to the study. Potentially eligible children were between 2 and 36 months of age at the time of study enrollment; had been hospitalized for at least 24 h with a suspected diagnosis of RGE; presented with an episode of three or more watery or looser-than-normal stools within a 24-hour period and/or 1 or more episodes of forceful vomiting; had not received a rotavirus vaccine in the past; were otherwise healthy (with the exception of RGE illness) with no significant medical history; and were not participating at the time of the study in any clinical trial for any drugs, vaccines or tests. Eligible parents/caregivers were those who served as the primary caregiver of an eligible child during RGE illness and were able to read and comprehend the local language (Taiwanese in Taiwan and Vietnamese in Vietnam) to provide a written informed consent. Professional caregivers were excluded.

The study was approved by the local IRB/EC at Mackay Memorial Hospital, Cathay General Hospital and Taichung Veterans General Hospital in Taiwan; and the local IRB/EC at HCMC Central Pediatric Hospital and by the HCMC Department of Health in Vietnam.

### Data collection

After the parents signed an informed consent, study investigators collected stool samples, which then were tested for the presence of rotavirus antigen using a commercially available assay (SD Bioline Rotavirus test, Standard Diagnostics Inc). A positive laboratory test determined the eligibility of a child. Study investigators recorded the rectal temperature and the percent dehydration of the child, collected information based on the reports of the parents, and utilized this information to assess clinical severity using the Clark and Vesikari scales [16, 17].

One parent per child was scheduled for a focus group session (or an in-depth ethnographic interview) at a later time, but within 30 days of the RGE episode, at a nearby field office in Taipei, Taiwan, or Ho Chi Minh City, Vietnam. Focus groups were held by professional ethnographers, and followed a semi-structured guide. The guide was developed based on a study conducted in the US and written in English [15], translated into the local language, back-translated and altered if necessary. Focus group topics were: description of the RGE episode, medical care for the illness, emotions and feelings during the illness, disruptions to the family routine, economic impact of the illness, and knowledge of RGE illness.

At the conclusion of the discussion, each parent/caregiver was asked to provide demographic information and to fill out a 37-item questionnaire to rank the importance of specified questions with respect to their recent experience with RGE illness. Parents indicated whether they felt that particular items affected them, and rated the importance of those items. The impact of items was calculated based on the proportion of patients who indicated each as important, multiplied by the mean importance score [ranging from 1 (least) to 5 (greatest)] indicated for this item (impact score = frequency cited × importance) [15].

### Data management and analysis

Each focus group was voice recorded, and notes were simultaneously taken. Audio files were transcribed, and the transcripts along with the notes served as the basis of content analysis. Data were extracted based on a priori questions of interest. Data summaries were analyzed to identify key themes. A combination of direct quotes and paraphrases was used to capture the themes of the interviews. Descriptive statistics of participant characteristics and the importance rankings were also produced. Contingency tables were used to describe frequency distribution for categorical variables, and means with corresponding medians were calculated for continuous variables. No statistical significance comparisons were made between Taiwan and Vietnam, due to the ethnographic nature of the study.

## Results

### Study participants

Altogether seven physicians in Taiwan and eight in Vietnam were contacted to participate as study investigators: all agreed to participate. In Taiwan and in Vietnam, respectively, 21 and 44 parents of potentially eligible children were approached, and 20 and 22 agreed to participate. In Taiwan, eight of the 20 were ineligible, and four parents were unable to participate in one of the focus groups at the designated location within 30 days. These four parents participated in in-depth interviews that followed the guide of the focus group sessions. In Vietnam, all 22 children were positive for rotavirus and all parents were available for and participated in the focus groups.

Altogether two focus groups and four in-depth interviews were conducted in Taiwan and four focus groups were conducted in Vietnam. The mean duration of focus group meetings/interviews was 120 min in Taiwan and 89 min in Vietnam.

#### Description of study participants

In Taiwan and in Vietnam, respectively, 12 and 22 rotavirus positive children and their parents were enrolled in the study. The demographic information of parents and their children can be found in Table 1. Information related to the RGE episode of the child can be found in Table 2. In both countries, the mean Clark and Vesikari scores indicated that most children’s condition was severe.

**Table 1:** Results: population characteristics

Characteristic	Vietnam N (%)	Taiwan N (%)
	or	or
	mean (SD)	mean (SD)
Age of the child with the RGE episode (in months)	15.27 (6.29)	25.08 (4.87)
Gender of the child with the RGE episode		
Male	17 (77.3 %)	7 (58.3 %)
Female	5 (22.7 %)	5 (41.7 %)
Age of the parent/caregiver (in years)	30.91 (6.61)	32.42 (5.20)
Gender of the parent/caregiver		
Male	4 (18.2 %)	1 (8.3 %)
Female	18 (81.8 %)	11 (91.7 %)
Marital status of the parent/caregiver		
Married	21 (95.5 %)	12 (100.0 %)
Educational level of the parent/caregiver		
High school or less	10 (45.5 %)	5 (41.7 %)
Some College	4 (18.2 %)	4 (33.3 %)
College Graduate	8 (36.4 %)	3 (25 %)
Type of occupation of the parent/caregiver		
Full time job	11 (50.0 %)	6 (50.0 %)
Part time job	4 (18.2 %)	0 (0.0 %)
Does not work outside home	7 (31.8 %)	6 (50.0 %)

**Table 2:** Results: Characteristics of RGE Episode

Characteristic	Vietnam N (%)	Taiwan N (%)
	or	or
	mean (SD)	mean (SD)
Number of days the child was in the hospital because of RGE	7.55 (5.08)	5.33 (2.46)
Who had to take a leave for the patient hospitalization?		
Nobody	8 (36.4 %)	6 (50.0 %)
Father	5 (22.7 %)	1 (8.3 %)
Mother	8 (36.4 %)	5 (41.7 %)
Caregiver (different from parents)	1 (4.5 %)	0 (0.0 %)
Number of working days lost due to the child hospitalization	9.14 (7.29)	2.83 (1.47)
Who will take care of the patient during the weekdays at home?		
Parent/caregiver	20 (90.9 %)	9 (75.0 %)
Another relative	2 (9.1 %)	3 (25.0 %)
Who had to take a leave for the patient care at home?		
Nobody	15 (68.2 %)	9 (75.0 %)
Father	5 (22.7 %)	2 (16.7 %)
Mother	2 (9.1 %)	1 (8.3 %)
Number of working days lost to take care of the ill child at home	4.71 (4.96)	2.00 (1.00)
(among those who stayed at home, *n* = 7)		(among those who took leave, *n* = 3)

#### Importance ratings of on the impact of rotavirus gastroenteritis

The items that were ranked highest by parents appear in Tables 3 and 4. They were similar in both countries, in that they were related to *the emotional aspects of the parent*, *logistical aspects*, and *the emotional well-being of the baby*. The lowest ranked items were similar in both countries, such as those that were in the context of *relationships to outsiders*, and *the clear-headedness and related emotions of the parents*. In Taiwan, however, *the mood of the baby* was ranked as low priority.

**Table 3:** Vietnam rank order survey top results

Rank Order	Item	Indicated importance	Rank Mean Score (on a scale of 1to 5)
1	I’ve been worried about my son/daughter	100.0 %	4.68
2	I had to get up during the night to see my son/daughter	100.0 %	4.32
3	S/He got tired easily	95.50 %	4.32
4	Have you had to spend time changing diapers because of diarrhea?	95.50 %	4.14
5	S/He was miserable	95.50 %	4.14
6	Traveling to the hospital is a strain	95.50 %	4.14
7	Did you have to stay in the house more than you wanted to?	100.0 %	4.05
8	S/He wanted someone with him all the time	95.50 %	4
9	Have you had to spend time trying to feed him/her rehydration solution?	90.90 %	4
10	S/He had very little energy	90.90 %	4
11	S/He wanted to be cuddled a lot	90.90 %	4

**Table 4:** Taiwan Rank Order Survey top results

Rank Order	Item	Indicated importance (yes/No)	Rank Mean Score (on a scale of 1 to 5)
1	I’ve been worried about my son/daughter	100.0 %	4.92
2	S/He wanted someone with him all the time	100.0 %	4.83
3	I had to get up during the night to see my son/daughter	100.0 %	4.75
4	Have you had to spend time changing diapers because of diarrhea?	100.0 %	4.67
5	S/He wanted to be cuddled a lot	100.0 %	4.33
6	S/He ate very little	100.0 %	4.17
7	S/He had very little energy	91.70 %	4.08
8	S/He got tired easily	83.30 %	4
9	I felt very tired	100.0 %	3.92
10	Have you had to spend time trying to keep his/her temperature down?	83.30 %	3.92

#### Focus groups and qualitative interviews

In Taiwan, eight parents participated in focus group discussions and four in in-depth interviews, and in Vietnam 22 parents participated in focus group discussions. Below are summaries of the discussions. Illustrative quotes of the parents are shown in Table 5 and 6.

**Table 5:** Illustrative Quotes Vietnam

Themes	Illustrative quotes
Describing the RGE episode	“It was something we never dealt with before. All the fevers before were easily taken care of with water and shower. But this disease is something totally different. The more we fed the baby with water, the more he vomited.”
	“Imagine my baby, 21 months, hyperactive and healthy, he runs around like an Energizer bunny. And one day, he just crashed like that. It’s a real shocker to us.”
Medical care for the illness	“The doctor’s method is absolutely opposite of what I expected. I expected the doctor should do anything to ease the disease immediately. But no.”
	“I often bring my kid to private clinic first, because big hospitals are very complicated and tiring process-wise”
Emotions and feelings during the illness	“We were so worried that the disease would come back we had to watch out everything my baby eats and drinks even after recovery. It became an obsession.”
	“When I saw those symptoms (RGE) in my baby, I was so afraid I could not find the strength to hold my baby, I could not even breathe”
	“The only moment when I first exhaled in relief, after days, was when my baby check-out of the hospital”

**Table 6:** Illustrative Quotes Taiwan

Themes	Illustrative quotes Taiwan
Disruption of family routine	“In particular while my child was hospitalized, we could not have regular social activity and has to rely on in-laws to help so we could take turns for care-giving.”
	“I also feel bad while I have to ask my mom’s help. She has to look after me as well as my child.”
Emotions and feelings during the illness	“My husband and I had the argument on how to look after the child. It has caused lots of pressure even on small issues, such as what is the right way to rinse the child after diarrhea.”
	“My daughter becomes insecure and would always cry in the middle of the sleep during midnight.”

### Describing the GRE episode

Parents in both countries described a similar array of RGE symptoms and behavior, such as moodiness, crying and the need to be cuddled. The most common symptoms described were abdominal cramps, vomiting up to 10 times a day, excessive amounts of liquid diarrhea, fever (as high as 40 °C), extreme fatigue and sleepiness, and weight loss. Parents reported that while they had experienced and dealt with most of these symptoms before, in RGE the array, the severity and the frequency of the symptoms were not only unprecedented but also frightening. Parents also reported potential disease transmission within their families and other social contacts, although this was not confirmed as rotavirus as part of this study.

### Medical care for the illness

#### Initial reaction to the illness

Initial reactions to the illness were similar in both countries. At the beginning when children were still more or less energetic, some parents thought the illness was mild and would pass quickly. They either just observed or tried traditional remedies to comfort the child. Most of the parents reported that they took their children to the hospital or the emergency room to prevent infecting other children, or when the sick child had become very weak and was confined to bed. Health care and hospital experience, however, was markedly different in the two countries.

#### Health care and hospital experience in Taiwan

Taiwanese parents believed that the children would recover soon due to the day-round presence of doctors and nurses. Parents reported that the children had to be hospitalized so that their condition would not become worse or to prevent getting infected with other pathogens. One child was discharged from the emergency room, only to be hospitalized later, while another one was re-hospitalized. According to the parents, stool tests were not performed in the emergency room whereas they were standard procedure during inpatient stays. The laboratory test gave the differential diagnosis from Enterovirus and Norovirus, which several parents reported to be epidemic at that time as well. Rehydration (either orally or intravenously), special protein-free and high-carbohydrate diet (which-as several parents reported-sometimes meant not letting the child eat anything at all for a day or two), fever medications, and (sometimes as per the request of the parent) anti-diarrhea medications were reported as the most common, and antibiotics and probiotics were occasionally prescribed. Overall, parents were satisfied with the results of the hospitalization, since their children’s condition improved as a result.

#### Health care and hospital experience in Vietnam

Many Vietnamese parents, especially in rural areas, first took their babies to nearby public or private clinics for outpatient care. The private clinics are preferred as they are viewed to have more competent staff. Some parents chose to take their children directly to the hospital bypassing the outpatient clinics altogether, and some were referred to the hospital by outpatient physicians. The hospital was viewed as the best place for medical services: care was round-the-clock, the facility was well-equipped, and hospital doctors were experienced and capable. Parents, however, were often unprepared for the experience of the large hospital setting, which was reported as crowded and uncomfortable. It was viewed that hospital doctors were more concerned about long-term treatment as opposed to quickly reducing the symptoms. Therefore, during the first days when the symptoms still persisted, parents became even more worried and desperate, and some purchased additional medication without the knowledge or consent of the treating physician. Miscommunication between parents and doctors regarding expectations for treatment and recovery led to some conflicts and contributed to the distress experienced by the parents.

### Disruption of family routine

The illness caused a considerable disruption of the daily routines of everybody in the nuclear family. During the illness, children were kept home from daycare and grandparents took care of them. Adults had to postpone or cancel their daily tasks to focus on and take care of the sick baby. Parents sometimes failed to tend to these other dependent family members: their meals were skipped, and their school activities were not adhered to. Those children who were placed on special diets were unable to dine with the family or consume familial foods and drinks. Families often had to cancel social activities and seek support from elderly family members. Some parents also reported that they or other family members also contracted the illness, thereby creating an even less manageable situation when a sick parent had to take care of a sick child. Caregivers reported high levels of distress within the families that led to frequent arguments among family members, especially concerning topics related to care giving and maintaining daily routines. In addition to the disruption of family routines during the illness, Taiwanese parents also complained about routine difficulties afterward (Vietnamese parents did not report on this aspect). For example, after the illness it took parents a long time to re-establish family routines and contacts with their social groups. Parents also reported greater attention to personal hygiene subsequent to the illness.

### Knowledge about RGE

#### RGE knowledge in Taiwan

Many of the parents in Taiwan had basic knowledge about rotavirus prior to their child’s illness, since their doctor or nurse had mentioned the rotavirus vaccine to them. They had thought though, that the vaccine was either not important or not effective, since if it was, then it would be compulsory. Their knowledge increased during the illness by being exposed to information in the hospital or looking it up on the internet. In one family, after the older child was diagnosed with RGE, the parents quickly sought vaccination for the younger child. One mother reported that the father believed that the illness was caused by playing outside too much, and wanted to restrict outside activities.

#### RGE knowledge in Vietnam

Many of the parents in Vietnam lacked knowledge about RGE, not only before but also after the episode, they were not aware of the etiology of the disease, and understood it to be a “diarrhea disease”. Parents were worried that the illness might impact their families and devastate them again, and felt ill-informed about how to prevent it from happening again. Upon hearing about the existence of a vaccine for rotavirus, many parents expressed surprise because they had not heard about it, and anger because the vaccination of the baby would have saved considerable mental and financial burden for the parents.

### Emotions and feelings during the illness

Parents expressed feelings in relation to several aspects of the illness: the illness itself and their experiences in the health care settings (in both countries), a control of emotions (in Taiwan), and the changes in their lives as a result of the illness (in Vietnam). The most often mentioned feelings in both countries were being scared, overwhelmed and shocked because of the severity of the RGE, and expressed that this had been the most traumatic illness in their lives. One mom in Taiwan reported feeling guilty, since she had known about the vaccine but did not have her baby vaccinated. Parents also felt inadequate to alleviate the suffering of their children. Since they were aware that serious dehydration could be life-threatening, they were also very worried, often for the life of the baby or for potential long-term detrimental effect on its health. One parent in Taiwan reported that she almost fainted when she saw her child’s skin becoming wrinkled as a result of severe dehydration. Another Taiwanese mother reported that several members of her family blamed other members of the family for the illness. Parents were also concerned that they might become sick as well and therefore jeopardize the care and the livelihood of the family. Parents started feeling relieved only when the child started accepting food. One Taiwanese mother reported anger and that she would no longer have contact with the family of the child that she suspected of transmitting to cirus to her child. Grandparents, on the other hand, did not initially view the illness as serious serious (thereby greatly frustrating the parents), but they did become more concerned as the illness progressed. Another source of frustration was when one of the parents or grandparents secretly fed the child despite the doctor’s order of not letting the child eat. One parent in Taiwan reported that when the child kept soaking the bed with liquid diarrhea she felt embarrassed to ask the nurse (again and again) to change the soiled sheet of her child.

#### Feelings in relation to health care settings—Vietnam

Parents in Vietnam, especially those who had only one child, expressed a great deal of emotions related to health care settings. They felt ill-informed, unprepared, and confused – feelings that were worsened by the lack of communication from the doctors in the hospital. When the doctor announced, that the baby had to stay in the hospital, many parents were shocked and believed that the condition had to be fatal. In addition, parents were distressed and frightened by the above-mentioned conditions in the hospital, and were worried that their children might contract other illnesses from coming into contact with other patietns. They experienced some relief when the children began to show some signs of recovery. Another source of great frustration, distress and unhappiness was how the life of the family changed due to the illness: daily routines were disturbed and considerable financial burden was inflicted on the family.

#### A control of emotions—Taiwan

An interesting aspect parents reported was a control of emotions. Both mothers and fathers reported controlling their emotions around their child or spouse, smiling a lot while they were with the child or spouse, and pretending that they were not worried about the illness. They, however, especially mothers, would cry a lot when they were alone. Female friends with children reportedly provided a great amount of emotional support mothers during the period of their child’s illness. Overall, however, mothers expressed more emotions and worries, while fathers were reportedly calmer during the illness. One father reported telling his child that pain would make a child stronger. Fathers felt they had the responsibility to suppress all their emotions so that their entire family did not panic. Therefore, some reported that the children would want to cuddle with the father instead of the mother, because they were calmer. Often, however, the relationship between parents got tense and they often fought with each other: mothers reportedly accused fathers of not doing everything for the child fast enough. In the end, both parents were mentally and emotionally exhausted.

### Economic impact of the illness

#### Economic impact—Taiwan

Most parents in Taiwan reported few expenses resulting from the illness, mostly because almost all participants had health insurance policies and much of the healthcare expenses were reimbursed by the insurance. Many families had their own cars, which facilitated transportation to and from the hospital. Expenses listed related to dining out (because of not having time to cook), an increased amount of diapers (some already toilet-trained children had to revert to using diapers again), taxi fares for going to the doctor or to the hospital (for those who did not have their own cars), the purchase of special formula because of the prescribed diet, and having to update health insurance in order to cover hospital expenses.

Many parents reported having to take annual leave to take care of the child, although they said that taking leave did not influence their income. Those who were at work reported that they had found it difficult to concentrate on their work, and were often on the phone with the grandparents to enquire about the child’s condition.

#### Economic impact—Vietnam

For the average Vietnamese family, illnesses result in considerable financial burden. This financial burden has two aspects: increased spending and decreased income. It was generally viewed that while the treatment itself might be inexpensive, the vast majority of costs for both the child and the caregivers stemmed from associated expenses, many of which were unplanned and/or unpredictable. For example, rural families who transported their children for treatment in the hospital incurred high costs due to the distance travelled, which, in some cases was as long as 2–3 h of driving. Costs included car rental, as well as accommodations and meals for the family for the duration for the hospital stay. Medical care costs associated with the hospital stay were also reported. Families who lived closer to the hospital incurred costs of travelling between home and hospital for visitations. To cover these costs, parents used savings, or in some cases incurred debt. In addition to the expenses, families often found themselves with decreased income, both short term and long term. For example, some parents needed to take unpaid leave. Moreover, they were afraid employers might lay them off due their prolonged absenteeism. This was especially a fear for parents in rural areas, where they said jobs were seasonal. Those who owned their own business reported they had to close their businesses while caring for the sick child.

## Discussion

This study assessed, in Taiwan and Vietnam, the impact of the rotavirus illness on the family among a group of parents whose children had recently been hospitalized for this illness. In both countries, the results indicate that families of children who had been hospitalized with rotavirus experienced, in many cases, a substantial emotional and economic burden as well considerable disruptions in family routines. There were, however, marked differences in certain aspects of the illness among Taiwanese and Vietnamese parents. Taiwanese parents reported satisfaction with the health care system, a great deal of effort to suppress emotions, a fair amount of knowledge about rotavirus, and negligible extra costs related to the illness. On the other hand, parents in Vietnam expressed concern about emotional well-being; many expressed negative feelings about their health-care experience, lacked knowledge about rotavirus, and were affected by a substantial financial burden due to costs associated with accessing medical care.

Similarly to the study findings by Mast et al. [15], this study also found that the emotional aspects of the illness were ranked as the top concern of parents. While the burden related to illness (such as direct and indirect medical and non-medical costs) and the burden on health may be measureable in monetary value, the emotional burden on both the parents and the children also exacts a cost that is difficult to quantify. Our findings are in line with the conceptual framework for health-related quality of life in acute gastroenteritis described by Johnston et al. [18]: they describe that both the emotional functioning (e.g. clinginess, irritability, sadness, etc); and the physical functioning concerning both symptoms (e.g. diarrhea, vomiting, lethargy, etc.) and daily activities (eating, sleeping, and normal routine) of the children were affected by gastroenteritis.

Another largely aspect of rotavirus illness reported by the parents was logistical problems related to managing the disruption of daily family routines. Johnston et al. [18] also describe the framework for parents in line with our findings: parent’s physical health and emotional and social functioning was impacted as a result of the illness. Similar to our findings, aspects of gastrointestinal illnesses described by previous studies include medications and therapeutics, daily adaptations, communications with health care professionals, uncertainty and loss of control [19, 20]. A multi-country study in Europe by Domingo at al. assessing the impact of RGE on the parents [21] described the highest impact among parents of children aged 12–23 months, mostly concerning symptoms and daily activities, with little difference among the countries.

Differences in the effect of rotavirus among Taiwanese and Vietnamese parents are noteworthy. One example is the aspect of health care Taiwanese parents reported satisfaction with health care system parents, many Vietnamese parents expressed negative feelings about their health-care experience. In addition, while Taiwanese parents reported negligible extra costs related to the illness, Vietnamese families were affected by a substantial financial burden as a result of RGE. These differences may be related to the different health care systems in Taiwan and Vietnam [22, 23]. The system in Taiwan is characterized by a national health care system that provides medical insurance for a majority of the population, whereas only about half of the population in Vietnam has health insurance.

Study limitations that should be noted include are related to sample size and generalizability. This was a qualitative study, with the goal to get an insight into the impact of rotavirus on the family, as opposed to hypothesis testing or the comparison of groups regarding quantitative measures. Children who participated in the study had had severe RGE and had been hospitalized. Parents of children whose rotavirus illness was not severe enough to warrant hospital treatment did not participate, and the study was not designed to generate insights into milder rotavirus illness. Only children admitted to Mackay Memorial Hospital, Cathay General Hospital, and Taichung Veterans General Hospital in Taiwan, and HCMC Central Pediatric Hospital in Vietnam were able to participate, and no clinics outside the capital were included (although many patients were from outside areas). Due to these limitations, the study may not be representative of all families affected by rotavirus in Taiwan and Vietnam, especially those with milder symptoms, or of minority populations within these countries. Parent’s perception of the impact of RGE is very subjective, so our findings represent their impressions and experiences as opposed to more subjective measures of effect [18]. Differences in cultural background (Taiwan vs. Vietnam and urban vs. rural) may be reflected in the perception of parents: while they may have the same experience, their perceptions of and reactions to these experiences may be different because their cultural background is different.

There are some available options for the treatment of rotavirus. Rehydration and supportive care may lessen to severity of the disease. However, disease prevention may only be achieved through vaccination. Vaccination programs are a cost-effective way to decrease both the humanistic and the economic burden of infectious diseases [24, 25]. There are currently two vaccines licensed in Taiwan: the pentavalent vaccine RotaTeq® by Merck & Co, and the monovalent Rotarix® by GlaxoSmithKline, but up to now their use has been limited to the private sector [26]. It has been estimated that universal vaccination in Taiwan would reduce RGE-related deaths, hospitalizations, emergency department, and outpatient visits by 91.7, 92.1, 83.7, and 73.4 %, respectively, and that from the healthcare perspective vaccination would be cost-neutral at a price per dose of 21.80 USD [26]. Rotavirus vaccines administered in Vietnam within the World Health Organization (WHO) Expanded Program on Immunization (EPI) have demonstrated good immune responses in infants [27]. Moreover, a multicentre, randomized, double-blind, placebo-controlled trial of infants conducted in Vietnam, Matlab, and Bangladesh also demonstrated the pentavalent rotavirus vaccine to be safe and efficacious against severe RGE [28]. A cost-effectiveness study on rotavirus vaccination in Vietnam found that introducing universal rotavirus vaccination in Vietnam would prevent 70 % of the outpatient visits, 84 % of the hospitalizations, and 83 % of the deaths that are associated with rotavirus infection [29]. This would translate to an overall 82 % savings – most of which would be in rural settings – of an estimated 5.3 million USD per year for all measureable costs. Other studies also confirmed that rotavirus vaccination would be a cost-effective health intervention in Vietnam [30, 31].

## Conclusions

Making rotavirus vaccination universally available for children in Taiwan and Vietnam would greatly reduce not only the measurable financial burden of rotavirus illness, but also the considerable psychological and emotional burden on the family that we found in this study.
